# Psychedelic-Assisted Psychotherapy—A Systematic Review of Associated Psychological Interventions

**DOI:** 10.3389/fpsyg.2022.887255

**Published:** 2022-06-10

**Authors:** Mauro Cavarra, Alessandra Falzone, Johannes G. Ramaekers, Kim P. C. Kuypers, Carmela Mento

**Affiliations:** ^1^Department of Cognitive, Psychological Science and Cultural Studies, University of Messina, Messina, Italy; ^2^Department of Neuropsychology and Psychopharmacology, Faculty of Psychology and Neuroscience, Maastricht University, Maastricht, Netherlands; ^3^Department of Biomedical, Dental Sciences and Morpho-Functional Imaging, University of Messina, Messina, Italy

**Keywords:** psychedelic-assisted psychotherapy, set and setting, theoretical models, psychedelics, review

## Abstract

Modern clinical research on psychedelics is generating interesting outcomes in a wide array of clinical conditions when psychedelic-assisted psychotherapy is delivered to appropriately screened participants and in controlled settings. Still, a number of patients relapse or are less responsive to such treatments. Individual and contextual factors (i.e., set and setting) seem to play a role in shaping the psychedelic experience and in determining clinical outcomes. These findings, coupled with data from literature on the effectiveness of psychotherapy, frame the therapeutic context as a potential moderator of clinical efficacy, highlighting the need to investigate how to functionally employ environmental and relational factors. In this review, we performed a structured search through two databases (i.e., PubMed/Medline and Scopus) to identify records of clinical studies on psychedelics which used and described a structured associated psychotherapeutic intervention. The aim is to construct a picture of what models of psychedelic-assisted psychotherapy are currently adopted in clinical research and to report on their clinical outcomes. *Ad-hoc* and adapted therapeutic methods were identified. Common principles, points of divergence and future directions are highlighted and discussed with special attention toward therapeutic stance, degree of directiveness and the potential suggestive effects of information provided to patients.

## Introduction

We are currently witnessing a growth in interest in psychedelic substances and their potential use for the promotion of mental health (Nutt et al., 2020). After an early phase set between the ‘50s and the ‘70s of the previous century when applications were being tested in psychotherapy (Abramson, 1960; Grof et al., 1973) and, more specifically, for the treatment of neuroses (Crocket et al., 1963), alcoholism (Smith, 1958; Leuner, 1967), end-of-life anxiety (Kast, 1967; Grof et al., 1973; Grof and Halifax, 1978), and chronic pain (Fanciullacci et al., 1977), psychedelic research almost got to a standstill. While modern clinical studies are obtaining promising results in some of the most difficult to treat psychiatric populations (for recent reviews see Wheeler and Dyer, 2020; Andersen et al., 2021), a portion of patients do not seem to benefit from psychedelic-assisted therapies (PAT) or end up relapsing (Nutt et al., 2020). While this issue may arise in part due to the limited number of sessions that are admissible in a clinical trial (Grof, 2016), such variability urges researchers to find ways to ways to increase response rates and the stability of clinical improvement.

Several authors hypothesized that the therapeutic effects of psychedelics cannot be explained by their pharmacological properties only (a model referred to as *psychedelic chemotherapy*; Pahnke et al., 1970) but rather that individual (“set”) and contextual (“setting”) factors play a pivotal role in some of the observed clinical gains with psychedelics (Leary, 1961; Pahnke et al., 1970; Hartogsohn, 2017). Classic psychedelics seem to increase neuroplasticity (Ly et al., 2018; de Vos et al., 2021; Hutten et al., 2021) and environmental sensitivity or suggestibility (Carhart-Harris et al., 2015; Carhart-Harris and Nutt, 2017; Carhart-Harris and Friston, 2019) both during the dosing session itself and in the following days (Majić et al., 2015). In a way, psychedelics seem to open a window of flexibility (Kuypers et al., 2016) that may relax higher level priors and increase sensitivity to bottom-up information (Carhart-Harris et al., 2018b; Carhart-Harris and Friston, 2019). In the context of psychotherapy, this could imply that patients are offered a window of opportunity to become more effective in modifying rigid behaviors, thought patterns, and emotional reactions and that psychotherapy itself must be carefully honed to take advantage of this fertile state.

Despite evidence and theoretical reasons supporting the importance of set and setting (Carhart-Harris et al., 2018b), we still lack a model identifying the specific factors on which to focus to maximize the effectiveness of PAT. Modern research still has to tackle the delicate matter of clearly describing the role of the therapist during dosing sessions and in the wider context of PAT. Current suggestions emphasize the importance of building rapport, “letting go” of resistance, promoting openness and reliance on unconscious processes in the context of a non-directive relationship (Johnson et al., 2008; Richards, 2015; Roseman et al., 2018). In the light of such considerations, the current review aims at collecting and describing the psychotherapeutic models that have been already used in clinical studies to provide a picture of current practices adopted by clinicians and researchers in the context of clinical research. Outcomes of such studies will be reported and common principles, points of divergence and future directions will be highlighted and discussed. All models will be presented using the name that the respective authors used to refer to them.

## Methods

**Figure 1:**
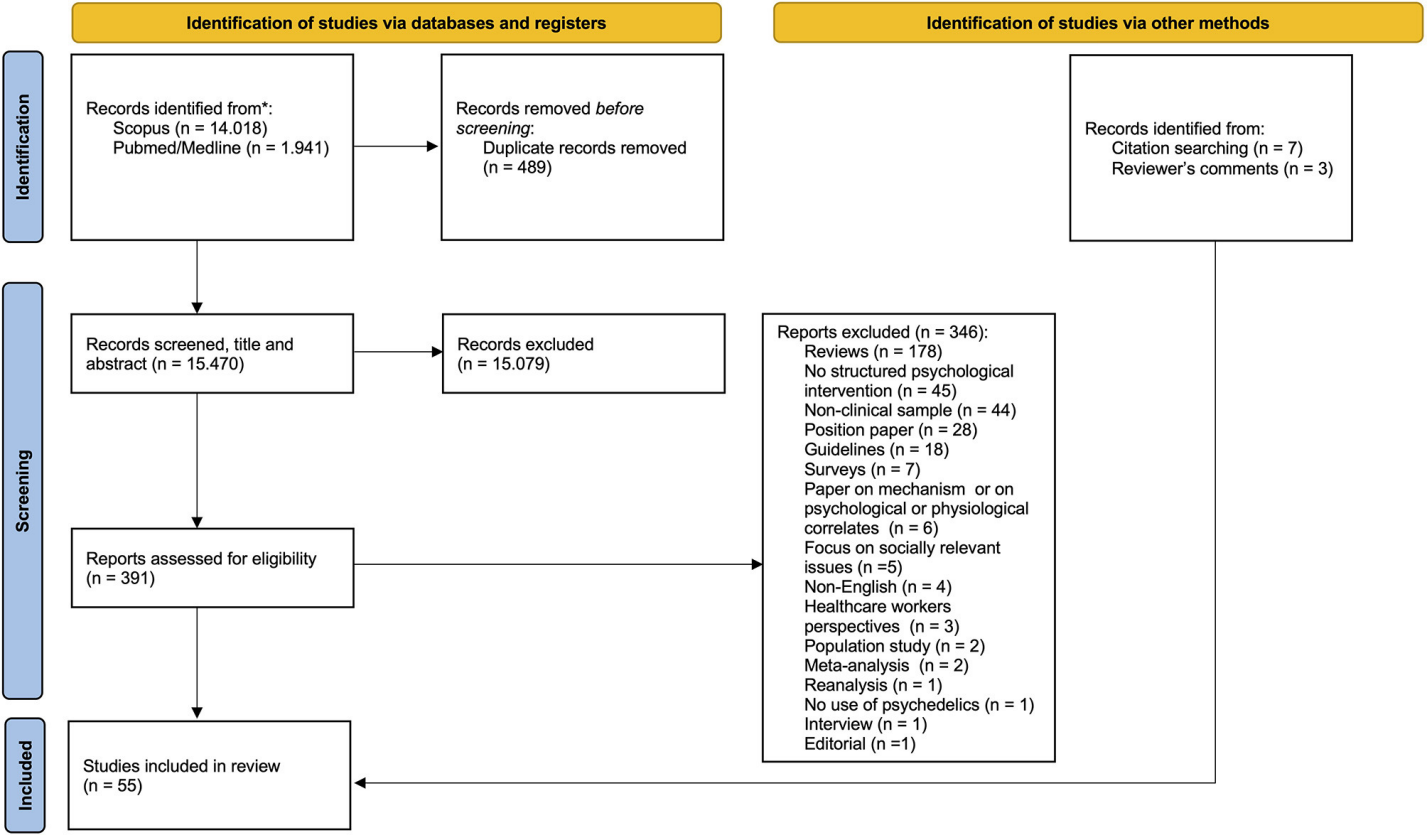
PRISMA flowchart.

We performed the following searches in Pubmed/Medline and Scopus databases: “psychedelic AND assisted AND psychotherapy,” “*substance name* AND assisted AND psychotherapy,” “psychedelic AND enhanced AND psychotherapy,” “*substance name* AND enhanced AND psychotherapy.” We restricted our search to clinical studies (i.e., which involved patients suffering from a psychiatric condition or psychological distress), written in English, which made use of a psychedelic or empathogen substance (i.e., LSD, psilocybin, MDMA, ayahuasca, ketamine, DMT, 5-MeO-DMT, mescaline), which contained a description of a structured associated psychological intervention and were published before September 2021. A total of 15.959 articles resulted from the search and 15.470 unique articles were identified. Two of the authors (M.C., C.M.) independently screened the articles to determine eligibility and when judgment differed discussions were held until consensus was reached. After screening titles and abstracts according to the above-mentioned criteria, 15.079 were excluded and 391 retained. Forty-five articles were retained after full-text screening, 7 were added after an iterative reference list search and 3 were added thanks to comments received during the paper review process. The resulting number of included papers is therefore 55 (Figure 1). We decided to organize the psychotherapeutic models in two main categories: *ad-hoc* therapeutic models (i.e., models were originally devised for use with psychedelics compounds) and adapted models (i.e., models that were devised in a more “traditional” psychotherapeutic setting and were later adopted for PAT).

## Results

We identified a total of 55 papers (Table 1) that reporting the results of a total of 26 studies in which the following therapeutic models were used: Cognitive Behavioral Conjoint Therapy (CBCT; *n* = 3; 1 open-label study), Cognitive Behavioral Therapy (CBT; *n* = 7; 1 case study, 2 open-label, 1 RCT), Human Relations Training Laboratory Group (HRTL; *n* = 1; 1 open-label), Ketamine Enhanced Therapy/Ketamine Psychedelic Therapy (KET/KPT; *n* = 5; 1 case study, 1 case series, 1 observational study, 2 RCTs), Medication-Assisted Psychotherapy (MAP; *n* = 7, 1 RCT, 1 pre-post, 1 case series), MDMA-Assisted Therapy plus Mindfulness Skills Training (MDMA-ATM; *n* = 1; 1 open-label), MDMA-Assisted Psychotherapy (MDMA-AP; *n* = 14; 1 case series; 1 small sample within-subjects, 3 RCTs, 1 pooled analysis of 6 RCTs), Motivational Enhancement Therapy (MET; *n* = 5; 1 open-label, 1 pilot RCT), Preparation Support Integration (PSI); (*n* = 7; 1 open-label), Supportive-Expressive Group Therapy (SEGT; *n* = 3; 1 open-label), Trauma Interventions using Mindfulness Extinction and Reconsolidation (TIMBER; *n* = 2; 1 RCT). In this pool, PAT's efficacy was tested in TRD, depression, opioid addiction, tobacco addiction, PTSD, AUD, issues related to life-threatening conditions, social anxiety in autistic adults and demoralization in HIV-infected patients. Notably, 45 papers were excluded because they did not provide a description of a structured psychological intervention. This was the case for studies which did not employ psychotherapy (e.g., Palhano-Fontes et al., 2019), used generic labels (e.g., Müller et al., 2020) or referenced guidelines for safety (e.g., Griffiths et al., 2016).

**Table 1:** Summary of included studies.

**References**	**Participants**	**Study design**	**Drug sessions**	**Type of psychotherapy**	**Control group**	**Primary timepoints**	**Primary outcome measures**	**Results**
Wagner et al. (2019); Monson et al. (2020), and Wagner et al. (2021)	6 couples	Pre-post	2, MDMA (75, 100 mg)	CBCT	None	End of treatment and 6-month follow up	CAPS-5, PCL-5, CSI	Improved symptomatology and couple satisfaction
Wilkinson et al. (2021)	40, TRD	RCT	28 responders to previous course of 6 ketamine infusion sessions (0.5 mg/kg) randomized to either CBT (*n* = 14) or (*n* = 14) TAU	CBT	TAU	14 weeks after last infusion	MÅDRS, QIDS	Significantly lower QIDS in CBT group. Significantly lower MÅDRS for the whole sample.
Ocker et al. (2020)	1, opioid addiction	Case study	5-day ketamine infusion (10 mg/h,0.09 mg/kg/h−70 mg/h, 0.6 mg/ kg/h)	CBT	None	30-day follow up	NRS, opioid use	Pain-free and no reported opioid use
Wilkinson et al. (2017)	16, TRD	Open-label	4, ketamine (0.5 mg/kg)	CBT	None	End of treatment and 12 weeks after last ketamine session	MÅDRS, relapse rate	Most relapses occurred after the completion of the CBT course. Ketamine non-responders did not seem to benefit from CBT.
Johnson et al. (2014, 2017), Garcia-Romeu et al. (2014), and Noorani et al. (2018)	12, tobacco addiction	Open-label	2 or 3, psilocybin (20 mg/70 kg, 30 mg/70 kg)	CBT for smoking cessation	None	6-month follow up, 12-month follow up	Biomarkers of smoking status and self-report measures of smoking behavior	80 and 67% abstinence
Bowen et al. (1970)	81, AUD; 59, AUD	Open-label clinical trial	1, LSD (500 or 25 μg)	HRTL	No drug or “low” dose.	1 year after end of treatment	Custom adjustment level measure	No significant differences.
Kolp et al. (2006)	~70, AUD	Case series	1 or 2	KEP	None	1 year	Abstinence rates	25–70%
Kolp et al. (2007)	2, LTI	Case study	1 (150 mg i.m.)	KET	None	End of life	Qualitative reports	Case 1: remission from panic attacks, relief from pain, discontinuation of painkillers. Case 2: no reported improvements.
Krupitsky et al. (2007)	59, heroin addiction	RCT, single vs. repeated ketamine doses	1 or 3, Ketamine (2.0 mg/kg im)	KPT	Single ketamine dose	1-year follow up	Abstinence rates	Greater abstinence rate in the repeated dose group
Krupitsky et al. (2002)	70, heroin addiction	RCT	1, ketamine (2.0 mg/kg im)	KPT	Ketamine, low dose (0.20 mg/kg im)	2-year follow up	Abstinence rates, Craving VAS	Greater abstinence rate and lower craving scores
Krupitsky and Grinenko (1997)	111, AUD	Non-randomized controlled trial	1, ketamine (2.5 mg/kg, im)	KPT	TAU	1-year follow up	Abstinence rate	Greater abstinence rate
Ross et al. (2016), Belser et al. (2017), Swift et al. (2017), Malone et al. (2018), and Agin-Liebes et al. (2020)	29, life-threatening cancer	RCT	1, psilocybin (0.3 mg/kg, p.o.)	MAP	Niacin	7, 6.5, 4.2 years	HADS, BDI, STAI	Improved depression and anxiety scores
Pahnke et al. (1969)	31, life-threatening cancer	Pre-post	1, LSD (200–500 μg)	MAP	None	End of treatment	Custom rating scale filled by external observers (e.g., relatives, clinical staff)	Improved scores of emotional and physical distress
Kurland (1985)	4, life-threatening cancer	Case series	1–4, LSD (100–400 μg)	MAP	None	End of treatment	Unstructured clinical evaluation	Improvement in mood, optimism and pain management
Danforth et al. (2018)	12, autistic adults	RCT	2, MDMA (75–125 mg)	MDMA-assisted therapy and mindfulness for adult autistic individuals	Inactive placebo	6-month follow up	LSAS	Improvements in social anxiety
Jardim et al. (2021)	3, PTSD	Case series	3, MDMA (75, 75, 125 mg)	MDMAAP	None	2 months after treatment	CAPS-4	Improvements in symptoms, depression Clinically significant CAPS score reductions; improvements in BDI, PTGI and GAF
Mithoefer et al. (2011, 2013, 2018, 2019); Oehen et al. (2013); Ot'alora et al. (2018); Barone et al. (2019), and Jerome et al. (2020)	105 (pooled), PTSD	RCT	2, MDMA (75–125 mg)	MDMAAP	Inactive placebo or active control (40 mg MDMA)	End of treatment, 12-month follow-up (minimum)	CAPS-4	Improvements in CAPS-4 scores between baseline and end of treatment and between end of treatment and follow up
Bouso et al. (2008)	6, PTSD	RCT	1, MDMA (50 or 75 mg)	MDMAAP	Inactive placebo	End of treatment	SSSPTSD, STAI, BDI, HAM-D, MSF, MS, SE/R	Reduced symptomatology
Mitchell et al. (2021)	90, PTSD	RCT	3, MDMA (80–120 mg each)	MDMAAP	Inactive placebo	2 months after treatment	CAPS-5, SDS	Reduced symptomatology and disability
Wolfson et al. (2020)	18, LTI	RCT	3, MDMA (125 mg)	MDMAAP	Inactive placebo	1 month after second MDMA session	STAI	Reduced trait anxiety
Sessa et al. (2019, 2021)	4, AUD	Pre-post	2, MDMA (187.5 mg for each session)	MDMAAP plus elements from MET and third wave CBT	None	9 months	PHQ-9, GAD-7, SADQ, SIP	Improved psychosocial functioning, reduced in alcohol consumption
Dakwar et al. (2020); Rothberg et al. (2021)	40, AUD	RCT, pilot	1, ketamine (*n* = 17; 0.71 mg/kg) or midazolam (*n* = 23; 0.025 mg/kg)	MET	Midazolam	21 days after infusion	Abstinence, time to relapse, heavy drinking days,	Significantly better abstinence rate, time to relapse, heavy drinking days in ketamine group
Bogenschutz et al. (2015), Bogenschutz et al. (2018), and Nielson et al. (2018)	10, AUD	Open label	1 or 2, psilocybin (0.3 mg/kg, 0.4 mg/kg)	MET plus preparation and integration	None	9-month follow up	Abstinence rates and % of drinking and heavy drinking days	Improved abstinence rates after psilocybin session (s) and reduced drinking and heavy drinking days
Carhart-Harris et al. (2016); Carhart-Harris and Nutt (2017); Watts et al. (2017); Erritzoe et al. (2018); Roseman et al. (2018); Stroud et al. (2018); Mertens et al. (2020)	20, TRD	Open-label	2, psilocybin (10 mg, 25 mg)	PSI	None	1 week, 3 and 6 months	QIDS	Improved depression at all timepoints
Anderson et al. (2020), Agin-Liebes et al. (2021), and Stauffer et al. (2021)	18, demoralization	Open-label	1, psilocybin (0.3 mg/kg)	SEGT	None	End of treatment and 3-month follow up	DS-II	Improved demoralization at the 3-month follow up
Pradhan et al. (2017, 2018)	20, PTSD	RCT	1, ketamine (0.5 mg/kg)	TIMBER	Inactive placebo	3 months after drug session	PCL, CAPS	Better and more sustained response

*AUD, Alcohol Use Disorder; BDI, Beck Depression Inventory; BDI, Beck Depression Inventory; CAPS, Clinician Administered PTSD Scale; CBCT, Cognitive Behavioral Conjoint Therapy; CBT, Cognitive Behavioral Therapy; CSI, Couples Satisfaction Index; DS-II, Demoralization Scale II; GAD-7, Generalized Health Questionnaire-7; GAF, Global Assessment of Functioning; HADS, Hospital Anxiety and Depression Scale; HAM-D, Hamilton Rating Scale-Depression; HRTL, Human Relations Training Laboratory; KET, Ketamine Enhanced Therapy; LTI, Life Threatening Illness; MDMAAP, MDMA-Assisted Psychotherapy; MÅDRS, Montgomery-Åsberg Depression Rating Scale; MAP, Medication Assisted Psychotherapy; MDF, Modified Fear Scale; MET, Motivation Enhancement Therapy; MS, Maladjustment Scale; NEO-PI-R, Revised NEO Personality Inventory; PCL, PTSD Checklist; PHQ-9, Patient Health Questionnaire-9; PSI, Preparation Support Integration; PTGI, Post-Traumatic Growth Inventory; QIDS, Quick Inventory of Depressive Symptoms; QIDS, Quick Inventory of Depressive Symptoms; RCT, Randomized Controlled Trial; SADQ, Severity of Alcohol Dependence Questionnaire; SDS, Sheenan Disability Scale; SE/R, Rosenberg Self-Esteem Scale; SEGT, Supportive Expressive Group Therapy; SIP, Short Inventory of Problems for Alcohol; SSSPTSD, Sever-ity of Symptoms Scale for Post-traumatic Stress Disorder; STAI, State-Trait Anxiety Inventory; TAU, Treatment As Usual; TIMBER, Trauma Interventions using Mindfulness Based Extinction and Reconsolidation; TRD, Treatment Resistant Depression*.

### *Ad-hoc* Therapeutic Models

#### Ketamine Enhanced Psychotherapy

Our search identified 5 studies investigating the efficacy of different versions of KET: 1 case series on AUD (Kolp et al., 2006), 1 case study on end-of-life distress (Kolp et al., 2007), 2 RCTs on heroin dependence (Krupitsky et al., 2002; Kolp et al., 2007), and 1 observational study on AUD (Krupitsky and Grinenko, 1997).

KEP, also sometimes referred as KPT or Ketamine Assisted Psychotherapy, was first introduced (albeit with a different name: affective contra attribution) in a 1992 clinical study on the treatment of alcoholism. It made use of other drugs in addition to ketamine (i.e., aethimizol and bemegride) during a single drug session to enhance emotional experiences and promote the consolidation of memories (Krupitsky et al., 1992; Krupitsky and Grinenko, 1997). Despite ketamine being a dissociative agent that does not act on 5-HT2A receptors as classical psychedelics do (Green et al., 2011), the authors often refer to it and to the experiences it produces as psychedelic. The model was developed in an attempt to integrate the establishment of aversive associations with a previously appetitive stimulus (i.e., aversive conditioning) with the shift in attitudes toward alcohol that psychedelic therapy seemed to promote in earlier studies (Krupitsky et al., 1992). In a later review article (Krupitsky and Grinenko, 1997), the authors describe this three-step approach in greater detail.

In the first stage, preparation is carried out over a 3-month period and aims at preparing patients by informing them about the different states of consciousness they will experience. An exploration of personal motives and goals for treatment along with beliefs concerning the cause of the disease is also carried out. Establishing positive expectations toward the outcome of the experience is considered another important part of preparation together with the co-construction of the therapeutic myth; the latter refers to a therapeutic narrative constructed by the therapist and the patient that could describe and explain the patients' suffering and hint at ways to resolve it (Frank and Frank, 1993). The session should integrate elements from the patients' personal life in an atmosphere of confidence and understanding. More specifically, patients are told that they may experience insights (e.g., on their personal problems, values, beliefs about themselves or the world) leading to adaptive changes in personality and that the psychedelic experience is instrumental in acknowledging the negative effects of alcohol abuse and the positive aspects of sobriety. Furthermore, they are instructed to fully surrender to the experience and are informed that the causes for their alcoholism are unconscious in nature, related to more central personality issues and that such causes will manifest themselves during the experience in symbolic form.

The second stage is the drug session, which is conducted by a psychiatrist and an anaesthesiologist. It includes the use of aethimizol (1.5% 3 ml, i.m.) to promote the consolidation of memory, bemegride (0.5% 1 0 mL, i. v.) to intensify emotional experience, and ketamine (2.5 mg/kg, i.m.) to achieve a psychedelic experience lasting between 45 and 60 min. During the session, new age/soothing instrumental music (Krupitsky and Grinenko, 1997; Krupitsky et al., 2002) is used with the intention to promote symbolic resolution of inner conflicts and facilitating cathartic experiences. Additionally, patients are exposed to so-called “psychotherapeutic influences,” i.e., therapist interventions that are “based on the concrete data of the patient's case history and [are] directed toward the resolution of the patient's personality problems and toward the formation of a stable orientation toward sobriety.” At the peak of the experience, while the therapist aims at shifting the focus of the experience toward the dysfunctional aspects of alcohol abuse (e.g., “Your fear is a result of vodka. It is vodka that has led you to the edge of the abyss”), patients are given the chance to smell alcohol to establish aversion toward the substance. Once the dissociative effects subside, the experience is discussed and interpretations are offered. Finally, patients are asked to write down a detailed report of the session (Krupitsky and Grinenko, 1997).

The third and final stage of KPT consists of a variable number of group psychotherapy sessions carried out the day after the drug session. In this context, the psychiatrist promotes integration *via* discussion, interpretation of the experience and consideration of lifestyle changes.

In the original 1992 paper, the authors employed this model to treat 86 male AUD patients and reported a remission rate of 69.8% at 1 year compared to 24% of the control group. KPT (in combination with existential psychotherapy) was also used to treat heroin addiction (Krupitsky et al., 2002) and was subsequently adapted to Ketamine-Enhanced Psychotherapy (KEP) to reportedly “extend it into another cultural context: the US” (Kolp et al., 2007). In this latter form, the treatment was used to treat end-of-life anxiety with the following structure: 3 weekly preparation sessions (75–80 min), one ketamine session (1–3 h) and a final integration session (75–80 min). KEP was also offered to patients suffering from alcoholism in two other formats: outpatient and intensive. The outpatient format consists of 3 preparation sessions, one of which is devoted to the formulation of a “psychospiritual” goal: a therapeutic objective that integrates the patients' personal motives for treatment, their goals for their future sober life and spiritual growth (Wolfson and Hartelius, 2016). By the latter, authors refer to a multitude of feelings, experiences and attitudes that usually concern feeling unity with others and the universe, increased chance of converting to a religion, experiencing a so-called “separation of consciousness from the body,” the feeling of being in contact with a higher power, the belief in the existence of “other dimensions or worlds that are parallel to ours” and similar other transpersonal phenomena. The treatment then proceeds with 1 ketamine session and 3 integration sessions (Kolp et al., 2006; Krupitsky et al., 2007). All integration sessions had the same structure and lasted 50 min; the ketamine session lasted for 3 h.

The intensive format had the same general structure of outpatient KPT and added daily all-day activity programs including psychotherapeutic and psychoeducation groups along with training in mind-body techniques. Initial data were collected in a clinical study in Russia in which the percentage of abstinent patients at 1 year post-treatment in a KPT group was compared with that of a control group (Krupitsky et al., 2007). The authors of the Russian study reported that 65% of patients in the KPT group (*n* = 111) remained abstinent compared to 24% (*n* = 100) in the control group. In the U.S., several adaptations of this model were created in an attempt to replicate results and progressively improve its efficacy even more (Kolp et al., 2006). In the first attempt the authors maintained the same structure of KPT as described above but condensed it in a 10-session, 10-week program which comprised 1 screening session, 4 preparation sessions, 1 goal-setting session, 1 ketamine session and 3 integration sessions. Contrary to expectations, this treatment led to an abstinence rate of only 25% in a group of 20 patients. Subsequently, the model was adapted to take place in a residential group setting and as such substituted the individual sessions with 30 weekly hours of psychoeducation, group activities, guided imagery exercises along with breathing, meditation, yoga, and cooking classes. The authors reported the results of 3 iterations of this model with each lasting, respectively, 1 week (35% 1-year abstinence rate, *n* = 15), 2 weeks (60% 1-year abstinence rate, *n* = 10), and 3 weeks (70% 1-year abstinence rate, *n* = 15). In all 3 iterations, patients with a history of psychedelic use were excluded at screening (Kolp et al., 2006). In summary, this treatment format progressively increased the 1-year abstinence rates over 1 (35%), 2 (60%), and 3 (70%) weeks of treatment.

In a double-blind, active-placebo controlled study, 70 detoxified patients suffering from heroin addiction were randomized into two groups employing KPT (Krupitsky et al., 2007). Both groups received 10 h of preparatory existential psychotherapy, 1 ketamine only infusion session (i.e., no other drugs were administered) and 5 more hours of integration psychotherapy. While one group received a high dose (2.0 mg/kg i.m.), the other received a low non-dissociative dose (0.20 mg/kg i.m.). Results showed improved abstinence, relapse rates, craving, and anhedonia in the high dose group while both groups showed improvements in anxiety, depression, internal locus of control, understanding of the meaning and purpose in life and spiritual development. The abstinence rate was about 85% at the 1-month assessment, about 18% at the 24 months follow-up and remained significantly higher for the KPT group over all timepoints. A subsequent study suggested that increasing the number of both ketamine (2.0 mg/kg i.m.) and integration sessions from 1 to 2 may lead to better abstinence rates at the 1-year follow-up (Krupitsky et al., 2007).

KEP was also used in two case studies (a success and a failure) involving a 62-year-old female and a 46-year-old male patients diagnosed with life-threatening illnesses and suffering from death-related anxiety (Kolp et al., 2007). The authors' intention was to highlight how certain patient characteristics may modulate treatment efficacy. While the female patient reported remission from panic attacks, discontinuation of morphine and lorazepam, increased feelings of comfort, the male patient did not resolve his death anxiety. This difference in clinical outcomes was attributed to the fact that while the woman was willing to discontinue benzodiazepine and decrease opiate use before the session, the man did not. They also pointed out that the woman never used psychedelics before while the man had an extensive history of psychotropic drug use. The authors of this paper seem to suggest that the efficacy of KEP is moderated by history of psychedelic use and they further hypothesize that the “novelty factor” associated with these substances may play a role in improving outcomes. Finally, they suggest that the concurrent use of other CNS depressants may diminish the patients' response to ketamine and potentially cause amnesia of the experience thus hindering therapeutic response.

In summary, across the literature KET is delivered in different formats. It proposes at least 3 preparation sessions, 1 drug session and at least 1 integration session. When administered in residential settings, it may be complemented by other activities and may include an additional drug session. Existential psychotherapy is the most cited model associated with the treatment and principles of behavioral therapy were incorporated in its original conceptualization. The role of therapists appears more directive compared with other models as clinicians are expected to actively work to establish positive expectations about the treatment, provide personality-related etiologies to explain AUD, and work to establish a structured therapeutic myth.

#### Medication-Assisted Psychotherapy

We identified 5 papers describing the efficacy of MAP in the context of a RCT (Ross et al., 2016; Belser et al., 2017; Swift et al., 2017; Malone et al., 2018; Agin-Liebes et al., 2020). MAP is a treatment model originally developed by Grof et al. (1973) and Grof and Halifax (1978) to be used in palliative care settings but that has been applied in the treatment of other conditions as well. Lysergic Acid Diethylamide (LSD) or Dipropyltryptamine (DPT) was administered in combination with psychotherapy to treat depression, anxiety and pain, and address existential issues in patients suffering from life-threatening illnesses. More recently, the model was used in conjunction with psilocybin administration (Ross et al., 2016; Belser et al., 2017; Agin-Liebes et al., 2020).

In its original structure (Grof et al., 1973; Grof and Halifax, 1978), MAP proposes 6–12 h of preparatory psychotherapy held over the period of 2–3 weeks focusing on the patients' present goals and issues instead of focusing on personal history and remote unresolved issues. Preparatory sessions are aimed at establishing rapport, working on family issues, addressing potential significant intrapsychic conflicts, confronting and accepting diagnosis, prognosis, and death. The overarching goal of the therapeutic model is to facilitate patients in living their days in the fullest and most meaningful way possible. Special attention is placed on preventing feelings of isolation and, therefore, the involvement of family members is encouraged. Once a good therapeutic relationship is formed and relevant issues are explored, patients are given more in-depth information concerning psychedelic sessions and the altered state of consciousness that they will experience. A session with the substance is then held by two trained facilitators in an appropriately furnished room where music can be played. Early accounts (Grof et al., 1973; Grof and Halifax, 1978) propose the use of family photographs to facilitate the resolution of relational problems and to promote the emergence of positive feelings.

More modern versions of MAP (Ross et al., 2016) encourage patients to bring items of personal and/or spiritual significance, to participate in acts (e.g., holding hands) aimed at conveying comfort and unity between the patient and the therapy team before the beginning of the session and to set their intentions toward finding relief from the psychological and existential suffering brought by cancer. During the course of the psychedelic experience, patients lie down wearing eye-shades and are invited to direct attention toward their internal experience. Therapists will remain present during the whole duration of the session and will offer non-verbal (e.g., holding hands, cradling, rocking) psychological and medical support when needed. During the final phases of the session, when the effects of the substance begin to subside, patients are encouraged to discuss their subjective experience in order to consolidate its memory. This phase was compared to psycholytic therapy (Crocket et al., 1963; Leuner, 1967; Gasser, 1996; Majić et al., 2015; Ross et al., 2016), an approach in use in the ‘60s in which patients would take smaller doses of psychedelics to facilitate the emergence of material during psychotherapy sessions. This process constitutes the first step of the subsequent integration work. Relatives can be invited during the termination period of the drug session. Integrative sessions, usually three, are held after each drug session and are meant to consolidate the memory of the experience and promote the processing of the emerged material.

MAP was recently used in a crossover RCT involving patients suffering from life-threatening cancer (Ross et al., 2016). The trial design involved a single psilocybin (or niacin) session (0.3 mg/kg, p.o.), three 2-h preparation sessions, three 2-h integration sessions and targeted depression and anxiety levels as primary outcomes. Psychotherapeutic work integrated elements drawn from evidence-based psychotherapies addressed to patients with life-threatening illnesses such as supportive psychotherapy, cognitive-behavioral therapy, existentially oriented psychotherapies, and psychodynamic psychotherapy. Results show that the treatment led to significant improvements in demoralization and hopelessness, spiritual wellbeing and quality of life. These changes occurred rapidly after the psilocybin sessions and were maintained at the 6.5 month follow-up. At that time, patients showed lower existential distress and better attitudes toward death. Response rates for depression were about 80% for the psilocybin group and between 20 and 30% (depending on the outcome measure) for the niacin group at the 1-day post session timepoint. Remission rates at the same timepoint were between 80 and 85% for the psilocybin group and between 20 and 30% for the niacin group. At the 7-week post session timepoint response rates were between 70 and 83% for the psilocybin group and between 14 and 40% in the niacin group. Remission rates at this timepoint were between 70 and 80% for the psilocybin group and between 20 and 40% for the niacin group. Response rates at the 1-day post session timepoint for anxiety were about 75% in the psilocybin group and about 40% in the niacin group. At the 7-week post session timepoint response rates were 58% for the psilocybin group and 14% for the niacin group. At the 6.5 months follow up, after the crossing over, response rates varied between 60 and 80% for depression and between 60 and 75% for anxiety. Long-term follow up (4.5 years) analyses of a subsample of patients from the above-mentioned study revealed that response rates were 57% for anxiety and ranged between 57 and 79% for depression. Remission rates ranged between 50 and 79% (Belser et al., 2017; Agin-Liebes et al., 2020). An earlier single sample, pre-post study (Pahnke et al., 1969) tested the efficacy of this model (using LSD, between 200 and 500 μg p.o. or dipropyltryptamine, between 60 and 105 mg i.m.) on a group of 31 cancer patients. Results showed “dramatic improvement” in 20% of patients, “moderate improvement” in 41.9% and no improvement in 22.6%. A very similar model, albeit labeled Psychedelic Peak Therapy (PPT), was described in a case series study (Kurland, 1985), which described the effects it had on 2 female patients suffering from breast cancer, 1 female patient suffering from pancreatic cancer and 1 female patient suffering from cancer to the uterine cervix. The authors reported improvements in all four patients in measures of mood, optimism and pain management.

In summary, MAP proposes 6–12 h of preparation, 1 drug session held by two trained facilitators and 3 integration sessions. The therapeutic model is eclectic, unstructured and integrates elements from multiple and diverse theoretical frameworks.

#### MDMA-Assisted Psychotherapy

Our search identified 12 papers (one of which analyzed the pooled results of 6 separate studies) describing the findings of investigations conducted on 6 separate samples (1 case series, 3 RCTs, 1 pooled analysis of 6 RCTs) that used MDMA-assisted psychotherapy for Post-Traumatic Stress Disorder (MDMA-AP) (Bouso et al., 2008; Mithoefer et al., 2011, 2013, 2018, 2019; Oehen et al., 2013; Ot'alora et al., 2018; Barone et al., 2019; Jerome et al., 2020; Wolfson et al., 2020; Jardim et al., 2021; Mitchell et al., 2021). Furthermore, we identified two papers that were based on the same small-sample within-subject study applying the model in an observational study on Alcohol Use Disorder (AUD; Sessa et al., 2019, 2021).

MDMA-AP (Mithoefer, 2016) is a structured approach to PAT that was developed by the Multidisciplinary Association for Psychedelic Studies (MAPS). MDMA-AP is an attempt to harness the pharmacological effects of MDMA to enhance psychotherapy effectiveness and aims at reducing or eliminating symptoms and achieving better functioning and wellbeing (Mithoefer, 2016). More specifically, the premise is that the substance-induced reduction in fear, increased interpersonal trust and positive emotions toward oneself and others will facilitate a corrective re-experiencing of the traumatic event in the context of a caring and supportive therapeutic relationship.

This treatment is based on earlier contributions by authors who proposed the use of psychedelics in psychotherapeutic settings (Pahnke et al., 1970; Greer and Tolbert, 1998), decisively relies on both preparation and integration sessions and adopts a seemingly non-directive, non-judgmental and empathetic approach to therapy (Mithoefer, 2016). The fundamental idea entails recruiting the patient's “inner healing intelligence”—i.e., “a person's innate capacity to heal the wounds of trauma” (Mithoefer, 2016)—to facilitate the processing of trauma through direct confrontation, mind-body techniques and a mindful and accepting mindset toward the MDMA-elicited internal experiences (Mithoefer, 2016). Therapists are encouraged to adopt a “beginner's mind”—i.e., an attitude that considers “any experience as an opportunity to heal and develop trust in their own inner healing intelligence”—while being receptive to potential hidden meanings and sources of insights emerging from the experience (Mithoefer, 2016).

To conduct MDMA-AP, therapists must undergo a specific training, should be proficient in administering treatments for PTSD, are encouraged to learn Holotropic Breathwork to gain experience with different states of consciousness, Internal Family Systems Therapy to learn how to work with multiple parts of the self, Sensorimotor Psychotherapy and/or mindfulness-based methods along with other breathing and bodywork techniques to help patients remain focused on the present experience and process trauma through their bodies (Mithoefer, 2016). Preliminary phases include the preparation of an appropriate physical setting and the adoption of mindsets and techniques that are thought to facilitate the therapeutic process on the therapists' part (Mithoefer, 2016).

The room in which the MDMA sessions will be held should be private, quiet, comfortable, well-furnished and allow for the presence of two therapists and music during the course of the experience (Mithoefer, 2016). Therapists are required to support patients through somatic manifestation of traumas, accept potential transpersonal experiences that may emerge during drug sessions and be ready to work with multiple parts of a patient's self. Such multiplicity is defined by the authors as a healthy phenomenon that “may be more pronounced in people who have experienced trauma” (Mithoefer, 2016). MDMA-AP places emphasis on preparation sessions as a means to determine patient eligibility, establish a functional therapeutic alliance, gather information about personal and trauma history, enquire about expectations, address concerns and prepare patients for the subsequent phases of the treatment (Mithoefer, 2016). Clinical studies usually include 2–3 preparation sessions each lasting 60–90 min (Bahji et al., 2020) which focus on personal history, therapeutic alliance and preparation of patients for the psychedelic session (Mithoefer, 2016). The general aim is to establish a sense of safety and comfort, an attitude of curiosity toward inner experience and trust toward one's inner healing intelligence (Mithoefer, 2016).

Unless medical or psychological contraindications are present, patients will take part in one or more drug sessions several weeks apart. MDMA sessions, 1–3 in clinical studies (Bahji et al., 2020), last 6–8 h and are facilitated by two co-therapists, preferably one female and one male (Mithoefer, 2016). Therapists are required to maintain an empathic presence and to seek a balanced attitude between keeping in mind the patients' intentions for the session and being open to the emergence of unexpected perceptions or memories, or ideas (Mithoefer, 2016). Therapeutic interventions are non-directive and encourage patients to adopt a similar attitude toward the experience (Mithoefer, 2016). In fact, they are advised to follow the spontaneous evolution of the experience and to trust their inner healing intelligence (Mithoefer, 2016). Painful memories, perceptions and ideas may emerge and, should therapists observe the adoption of avoidant attitudes on the patients' part, they should encourage them to mindfully confront such material and advise them on techniques they can use to work through the emotional distress (Mithoefer, 2016). Regular check-ins with silent patients are recommended in order to learn about their psychological state and experience (Mithoefer, 2016). During the later part of the session, while the effects of the substance begin to subside, therapists are advised to ask whether the patient is willing to provide more details about their experience (Mithoefer, 2016). These may be further discussed during follow-up (integration) sessions (Mithoefer, 2016). Patients are accompanied until the end of the session and are helped in resolving any potential emotional or physical distress that may persist (Mithoefer, 2016). Once the session is over, patients may be offered to spend the night in the treatment facility where their mental and physical state will be monitored by the clinical staff (Mithoefer, 2016). They can invite a significant other in. The morning after the MDMA session, the first integration session takes place (Mithoefer, 2016).

Integrative sessions-−2–3 in clinical studies (Bahji et al., 2020) aim at integrating the experience in the patients' ongoing therapeutic process and everyday life and usually last 60–90 min (Mithoefer, 2016). MDMA-AP rests on the assumption that the effects of the treatment will continue to unfold between sessions and after the treatment program is complete (Mithoefer, 2016). Patients are therefore informed that new insights, improvements in resilience, emotional wellbeing and relational skills may keep occurring (Mithoefer, 2016). Furthermore, should they experience psychological problems or suffering as a result of the MDMA-induced experience, integration sessions can be used to tackle such issues and define the therapeutic trajectory (Mithoefer, 2016). In this phase, therapists answer patients' questions, adopt a supportive and validating attitude toward the experience and encourage them to process new insights concerning their trauma, history, relationships and personal life (Mithoefer, 2016). The non-directive character of the therapy is maintained through the integration phase and while interpretations by the therapist are allowed, they “should be minimized” (Mithoefer, 2016). Integrative work begins the morning after the first MDMA session (Mithoefer, 2016). Patients are invited to discuss their experience in greater detail. Therapeutic work may be complemented by focused bodywork (e.g., “giving resistance for the participant to push against;” Mithoefer, 2016), breathing techniques and other approaches to facilitate the processing of the emerged material (e.g., trauma-related memories and physical sensations) through arousal regulation (Mithoefer, 2016). Before the closing of the treatment program, patients' potential concerns and questions must be addressed and the techniques and procedures that they found useful can be reviewed and consolidated.

MDMA-AP has been tested in several studies investigating its potential in the treatment of PTSD in various populations. A recent review and meta-analysis reported high rates of clinical response (72%) and more than 2 times the probability of achieving remission in the experimental groups with large effect sizes in symptom reduction at long term follow-up (between 12 and 74 months; Bahji et al., 2020). A study which tested this model plus elements from third wave CBT and MET as a potential treatment for AUD reported that in a sample of 14 previously detoxified patients, an 8-week course of MDMA-AP led to a significant drop in average units consumed per week from baseline (130.6 units/week) to the 9 months follow up (18.7 units/week) in 11 patients (79%) with 9 patients (64%) who were completely abstinent (Sessa et al., 2021). Finally, MDMA-AP was used to treat anxiety associated with life-threatening illnesses in a sample of 18 patients in a randomized pilot study (Wolfson et al., 2020). While the MDMA-AP group reported greater reductions in anxiety, the difference failed to reach the significance threshold.

In summary, MDMA-AP proposes 2–3 preparation sessions, 1–3 drug sessions held by two facilitators (preferably a male and a female) and 2–3 integration sessions per drug session. The therapeutic model places emphasis on non-directiveness, provides suggestions that highlight the role of the inner healing intelligence (the construct considered responsible for clinical change by the authors), aims at establishing positive expectations about the treatment and enquires about those of patients, allows for therapist direction of the session in case avoidant tendencies are observed and encourages interpretation and search for hidden meanings on the therapist side. Furthermore, bodywork techniques, mindfulness and sensorimotor psychotherapy are considered valuable tools to work through the physical manifestations of trauma. Given that the patients' psyche is conceptualized as made of several parts, elements from Internal Family Systems therapy are used throughout all phases of treatment.

#### Preparation, Support, Integration

Our search identified 7 papers describing the findings of 1 open-label feasibility study employing the PSI model to treat patients suffering from Treatment Resistant Depression (TRD) (Carhart-Harris et al., 2016, 2018a; Watts et al., 2017; Erritzoe et al., 2018; Roseman et al., 2018; Stroud et al., 2018; Mertens et al., 2020).

PSI (Carhart-Harris et al., 2018a) is a psilocybin-assisted model to treat TRD. The model is briefly outlined in two papers (Carhart-Harris et al., 2016; Stroud et al., 2018) describing the outcomes of an open-label feasibility study. It comprises one 4-h preparation session aimed at building a trusting relationship and encouraging patients to talk about their personal history and their hypotheses concerning the origin of their depression. They receive information concerning psilocybin's psychological effects and take part in a drug-free simulation of the psychedelic session. The two dosing sessions (10 and 25 mg, p.o.) take place 1 week apart and are held in a pre-decorated room in which patients can lie supine wearing eye shades and listen to music. Two psychiatrists supervise the session and adopt a non-directive approach. The aim is to facilitate an “uninterrupted journey” for patients while regularly performing check-ins to keep track of their physiological and psychological state. Once the first session is over, patients are taken back home by a close friend or a relative and are contacted *via* telephone the next day to evaluate their wellbeing and monitor for adverse effects. Two, 3, and 5 weeks after the second dosing session, email assessments of clinical progress are performed. One final follow-up is performed remotely 3 months after the last high-dose psilocybin session. In another description, the model also includes an integration session held the day after dosing in which patients are invited to talk about their experiences and are met with compassionate listening and occasional interpretations to promote and consolidate positive change (Carhart-Harris et al., 2018a; Stroud et al., 2018).

Results reported in the open-label TRD papers show improvements in severity of self-reported depressive and anxiety symptoms 1 week, 3 months (*n* = 12), and 6 months (*n* = 19) after dosing (Carhart-Harris et al., 2016, 2018a). Remission rates were 67% at 1 week and 42% at 3 months while response rates were 58% at 3 months and 31% at 6 months. The 6-month follow-up study also reported decreases in suicidality at 1 and 2 weeks post-treatment (Carhart-Harris et al., 2018a). Further evidence shows that psilocybin and PSI improve processing of emotional faces in TRD patients (Stroud et al., 2018) and that clinical improvements seem to be predicted by the quality of the psychedelic experience (Roseman et al., 2018). Finally, the authors reported significant decreases in neuroticism and increases in extraversion and openness to experience (Erritzoe et al., 2018).

In summary, PSI proposes one 4-h preparation session, 1 drug session held by two psychiatrists and 1 integration session. The theoretical model is broadly described as non-directive and the therapists take an active role during preparation in gathering the hypotheses that participants have concerning the origin of their depression. During the integration phase, therapists are required to offer compassionate listening as well as provide interpretations and facilitate the consolidation of positive change.

#### Trauma Interventions Using Mindfulness-Based Extinction and Reconsolidation

Our search identified 2 papers investigating the efficacy of TIMBER on a single sample in the context of a RCT (Pradhan et al., 2017, 2018). TIMBER is a psychotherapeutic model conceived to treat PTSD (Pradhan et al., 2017) through the application of the principles of extinction, reconsolidation and arousal regulation. It employs mindfulness techniques and was used to improve the stability of clinical gains that were obtained in a previous study employing ketamine as a therapeutic agent (Feder et al., 2014). TIMBER, in its short version, is administered in the course of a 60-min period and begins with a 10-min reactivation of traumatic memories (Pradhan et al., 2017). More specifically, patients are asked to recall a traumatic event through a pre-prepared personalized script with the objective of activating arousal responses for the following 2–3 min. The 40-min infusion period with 0.5 mg/kg (R, S)-ketamine is then initiated and patients are asked to recall calming memories and to practice the STOPP [Stop, Three mindful breaths, Observe, Practice more, Proceed (Pradhan and Pinninti, 2016)] mindfulness procedure for 5 min to de-escalate emotions such as frustration, anger, anxiety and reduce impulsivity. The goal of this phase is to achieve a state referred to as mindfulness-based detached monitoring.

In a small placebo-controlled, cross-over study (Pradhan et al., 2017), 10 patients with chronic and refractory PTSD were randomized in two groups to receive TIMBER with ketamine (dose, i.v.) or placebo (saline solution). The first session of both programs was the infusion session followed by 11 non-infusion sessions of which two were held in the same week of the infusion while the rest took place on a weekly basis. The psychotherapeutic model for the remaining 9 sessions was the same as that detailed above. Findings showed that before cross-over 9 out of 10 participants reported improvements in PTSD symptoms, depression and anxiety measures independent of group allocation. No significant differences in the magnitude or duration of treatment effects were observed between the two groups. A subsequent extension of the study including 10 additional patients demonstrated a statistically significant 108% increase in response duration (18 days on average) after TIMBER with ketamine compared to TIMBER with placebo (Pradhan et al., 2018).

### Adapted Therapeutic Models

#### Brief Supportive Expressive Group Therapy

Our search identified 3 papers on 1 open-label study employing psychedelic-assisted SEGT to treat demoralization in a sample of demoralized older long-term male AIDS survivors (Anderson et al., 2020; Agin-Liebes et al., 2021; Stauffer et al., 2021). SEGT is a model that was created in the context of palliative care (Classen et al., 1993) and was adapted for use with HIV-infected patients (Maldonado et al., 1996). As described in SEGT manual (Maldonado et al., 1996), the treatment aims at creating an environment of mutual support to contrast the feelings of isolation that are often a consequence of receiving this diagnosis (Maldonado et al., 1996). Patients are encouraged and helped in disclosing their condition to their loved ones and in learning how to ask for the support they need (Maldonado et al., 1996). This is especially relevant when it comes to intimate relationships and importance is placed into promoting conscious and safe sexual practices (Maldonado et al., 1996). Cultivating openness and expression of both positive and negative emotions is a primary goal of the treatment in order to relieve and encourage patients in their personal journeys (Maldonado et al., 1996). Emphasis is given in integrating the changes in self and body image (Maldonado et al., 1996). In fact, patients may find themselves in the position of requiring help in everyday activities for the first time in their lives and such experience may be troubling for some (Maldonado et al., 1996). Furthermore, the onset of opportunistic infections and other related pathologies may lead to changes in appearance that may need therapeutic work to facilitate acceptance and adaptation (Maldonado et al., 1996). After the diagnosis, patients find themselves tackling new challenges in life and, to support them, SEGT strives to expand and improve their coping skills through the experience of their fellow group members and therapist contribution (Maldonado et al., 1996). The development of a life project is encouraged and exploration of life values is intended as a tool to help clarify personal goals (Maldonado et al., 1996). Self-hypnosis is taught as a way to manage pain, improve sleep quality and deal with stress (Maldonado et al., 1996). Given the central role that pharmacological therapy has in the management of the infection and the high risk of poor adherence to treatments, patients are encouraged to be actively involved with the medical staff to promote a good doctor-patient relationship (Maldonado et al., 1996). Finally, receiving the HIV diagnosis may be the first time in which patients are confronted with their own mortality and therapists are instructed to address death related issues, avoidance behaviors and patterns of thoughts to allow group members to explicitly talk about death (Maldonado et al., 1996). Throughout the duration of the treatment, interaction between group members is actively pursued and the focus of the discussion is kept on personal and concrete issues related to HIV/AIDS in a climate of empathy and unconditional positive regard (Maldonado et al., 1996). SEGT sessions are 90 min long and are conducted by two co-therapists who have experience in working with patients with HIV (Maldonado et al., 1996).

SEGT was used as an adapted model in conjunction with psilocybin sessions in studies aimed at testing its effectiveness in treating demoralization in male older long-term AIDS survivors (Anderson et al., 2020). This integration model included a 90-min psychotherapy session held before group initiation in which patients received psychoeducation on group therapy and psilocybin. Patients underwent 12–15 h of group psychotherapy over the course of 7 weeks and took part in 1 8-h individual psilocybin session (0.30–0.36 mg/kg p.o.). Finally, a second 2-h individual psychotherapy session was held the day after the psilocybin session. The self-hypnosis module of SEGT was replaced by breathing and mindfulness exercises with the intention of providing patients with techniques they could use during dosing sessions. Results showed clinically relevant change in demoralization (Anderson et al., 2020) and significant reductions in self-reported attachment anxiety at the 3-month follow-up (Stauffer et al., 2021). Regarding response rates, 88.8 and 66.7% of participants achieved a 2-point improvement in demoralization at treatment end and follow-up, respectively. Fifty percent and 33.3% of them more than halved their baseline demoralization scores at treatment end and follow-up, respectively. An interpretive phenomenological analysis conducted by the same authors (Agin-Liebes et al., 2021) suggested that patients felt freed from their avoidant tendencies, able to access and process painful and self-transcendent feelings. They also reported an increase in prosocial attitudes and positive emotion along improvements in meaning-making and post-traumatic growth.

In summary, psychedelic-assisted SEGT integrated the psychedelic preparation in the preliminary session normally devoted to psychoeducation on group therapy. The treatment includes 1 psilocybin session and a subsequent integrative session. SEGT emphasizes the importance of tackling specific topics common to HIV-infected patients in group through direct confrontation with themes, emotions and practical issues in an atmosphere of openness and acceptance.

#### Cognitive Behavioral Therapy

Our search identified 7 papers about the efficacy of psychedelic-assisted CBT. These regarded tobacco dependence (4 papers, open-label study: Garcia-Romeu et al., 2014; Johnson et al., 2014, 2017; Noorani et al., 2018), opioid dependence (1 paper, case study: Ocker et al., 2020) and TRD (2 papers, 1 RCT: Wilkinson et al., 2021); 1 open label study: (Wilkinson et al., 2017). CBT in conjunction with psilocybin has been used to support opioid tapering (Ocker et al., 2020) and, in the form of the Quit For Life programme (QFL) (Marks, 1993), to treat tobacco addiction (Garcia-Romeu et al., 2014; Johnson et al., 2017). Furthermore, it has been employed with ketamine to treat TRD (Wilkinson et al., 2017).

QFL is a self-help CBT-model designed to achieve smoking cessation in 7–10 days and provides patients with tools to prevent relapse (Marks, 1993). The program frames smoking as a “psychological addiction,” asserts that quitting can be achieved with a moderate amount of willpower, poses emphasis in increasing patients' self-efficacy and offers 30 different CBT-based approaches to reach such a goal. QFL is administered in the form of a handbook, cards, charts and audio recordings containing a summary of the material, relaxation music and suggestions to promote abstinence. Once a date for actual cessation is set, the first phase of the treatment begins: reduction. At this stage, patients are encouraged to reduce smoking by 50% everyday and are expected to reach abstinence 1 week later. Relapse prevention is the second phase of the program and makes use of several techniques such as mental imagery, suggestion, meditation and relaxation while teaching how to deal with other smokers. The program was initially tested in a two-year follow-up study (Sykes and Marks, 2001; Marks and Sykes, 2002) and authors reported that about 28% of individuals achieved abstinence or significant reductions in the QFL group while only about 6% did in the control group. The difference between groups was statistically significant.

In an open-label pilot study recruiting 15 nicotine-dependent smokers, Johnson et al. (2014) tested the effects of a 15-week treatment program which integrated QFL with psilocybin sessions (2014). To this aim, they structured a model in which participants first established a target quit date (TQD) 5 weeks from the beginning of treatment and then received 4 weekly QFL inspired sessions. Each session also conveyed information about psilocybin, contained a 10-min relaxation exercise along with the smelling of a scented oil and a guided imagery exercise, two elements drawn from another manualized smoking cessation intervention (Zernig et al., 2008). The first psilocybin session took place in week 5, employed an oral 20 mg/70 kg dose and coincided with the TQD. Following the first administration, participants went on with the weekly QFL program and took part in a second 30 mg/70 kg psilocybin session at week 7. Participants who still had not quit by that time, were offered a third psilocybin session at week 13 (30 mg/70 kg, unless the participants themselves wished to lower it) while weekly QFL sessions went on until week 15. During all psilocybin sessions participants were asked to state a previously elaborated motivational statement for smoking cessation. They lay on a couch while listening to music through headphones, received support from the research staff and were involved in a guided imagery exercise during the final phases of the session when the effects of psilocybin were subsiding. Finally, they were asked to write down a description of their experience to be used for discussion in an integration session that was held the following day.

In summary, psychedelic-assisted CBT for smoking cessation proposes 4 QFL/preparation sessions which included relaxation, guided imagery and smelling scented oil, 2 or 3 drug sessions held by at least one staff member and 1 integration session per each drug session. At the 6-month follow up, the authors reported significant reductions in daily smoking compared to baseline and that 80% (12 out of 15) of participants showed 7-day point prevalence abstinence. At 12 and ≥16-months post-treatment, 67% (10 of 15) and 60% (9 of 15) were confirmed abstinent, respectively (Johnson et al., 2017). Subsequent qualitative analysis of participants' accounts revealed that they experienced insights concerning their reasons for smoking, increased feelings of interconnectedness, awe and curiosity and long-term positive changes in pro-social behavior and aesthetic appreciation (Noorani et al., 2018). Abstainers scored higher on measures of psilocybin-occasioned mystical experiences (Garcia-Romeu et al., 2014).

Ketamine plus CBT for opioid tapering was used in a recent case-study in which a 55-year-old male patient suffering from complex regional pain syndrome treated with opioids for 12 years achieved abstinence after a 5-day ketamine infusion program while continuing a psychotherapy initiated 3 years prior (Ocker et al., 2020). Infusion began at 0.09 mg/kg/h and was titrated twice daily in 0.09 mg/kg/h increments. On the last day the patient had the dose halved in the morning and then discontinued in the afternoon.

Another ketamine plus CBT intervention was used in a study aiming at extending the stability of the antidepressant effect of the substance (Wilkinson et al., 2017, 2021). To this aim, the authors recruited 48 TRD patients and treated them with 6 intravenous infusions of ketamine (0.5 mg/kg over 40 min) during the course of 3 weeks. The 28 (58%) participants who showed an improvement >50% in depression scores were classified as responders and randomly assigned to two groups: 14 weeks of CBT or treatment as usual (TAU; regular visits with physician). The Quick Inventory of Depressive Symptoms scores showed more sustained improvement in the CBT group compared to TAU while the Montgomery Åsberg Depression Rating Scale scores did not reveal effects.

The CBT used in this study was based on Beck's model and comprised psychoeducation, cognitive restructuring and behavioral activation. The model included no preparation sessions and no formal integration sessions. Once the infusion cycle was completed, patients were referred to certified CBT therapists.

#### Cognitive Behavioral Conjoint Therapy

Our search identified 3 papers on an observational study testing the efficacy of psychedelic-assisted CBCT in the treatment of couples in which one member suffered from PTSD (Wagner et al., 2019, 2021; Monson et al., 2020). CBCT (Monson and Fredman, 2012) is a treatment model for PTSD which, instead of focusing on patients alone, recruits their intimate partners in an attempt to improve treatment outcomes. In this context, PTSD is conceptualized as an interpersonal disorder given (a) the evidence showing that symptom severity correlates with intimate relationship adjustment and aggression (Taft et al., 2011); (b) that the construction of meaning after the traumatic experience is an interpersonal process (Monson et al., 2010); and (c) that the functioning of the relationship itself and of the intimate partner influences PTSD severity (Bradley et al., 2005; Lambert et al., 2012; Shnaider et al., 2014). CBCT promotes the development of new skills in the dyad as a whole and considers the relationship itself the actual client of the treatment (Monson and Fredman, 2012).

The efficacy of CBCT was tested in military samples in both controlled and uncontrolled studies (Liebman et al., 2020). This 15-session program comprises three phases synthesized by the acronym R.E.S.U.M.E. Phase 1 (*R*ationale and *E*ducation) provides psychoeducation concerning PTSD and its relational consequences and aims at building a climate of physical and emotional safety through behavioral strategies intended to increase positive affect and behaviors. Phase 2 (*S*atisfaction Enhancement and *U*ndermining Avoidance) focuses on teaching and training communication skills and treating emotional numbing and behavioral avoidance. The goal is to promote the approaching of feared situations and increasingly engage the dyad in enjoyable activities. Finally, in phase 3 (*M*eaning Making and *E*nd of Therapy) the goal shifts to trauma related appraisals and its consequent current cognitions. The aim is to weaken factors that consolidate PTSD symptoms and relational problems. A recent review shows that CBCT leads to significant improvements in PTSD symptoms, comorbid conditions and relational satisfaction in both clinician and patient ratings (Liebman et al., 2020).

CBCT was recently used in an uncontrolled trial aimed at testing its effectiveness in six couples with the inclusion of two 6-h MDMA sessions (Monson et al., 2020) conducted in accordance with the guidelines specified in an earlier work (Wagner et al., 2019). The first two sessions of the treatment covered the R and E sections and were held on day 1. The next day participants would go through two more sessions covering part of the S and U sections. During the same day they would take part in the first dosing session (75 mg MDMA; 90 min after administration, patients could decide whether they wished to take an optional supplemental half-dose). The rationale was that by that time the couple would have already developed the necessary skills to effectively communicate during the session and share their experience. The first MDMA session was followed by an integration session on day 3 and over the subsequent 3 weeks participants would complete the S and U sections. The second MDMA session (100 mg plus an optional 50 mg) would then be held on day 23, to take advantage of the increased focus on trauma processing that the S and U sections entail. The program then proceeded with an integration session and the completion of modules M and E. All MDMA sessions took place in a room where participants could lie on a reclining chair. They listened to music through headphones, wore eyeshades and were encouraged to “spend time inside.” Results showed significant and sustained improvements in clinician-assessed PTSD with almost all participants achieving remission at the 6-month follow up. Five patients out of 6 showed remission at post-treatment follow-up (1 and 6 months). Depression, emotion regulation, trauma-related beliefs along with patient and partner satisfaction also improved. Subsequent analyses revealed improvements in post-traumatic growth measures, relational support, social intimacy as well as greater wellbeing within the couple, improved psychosocial functioning and empathic concern (Wagner et al., 2021).

In summary, psychedelic-assisted CBCT relies on a structured treatment model, does not include formal preparation sessions and places 2 drug sessions once certain communication skills are sufficiently developed. The drug sessions involve both partners and are followed by an integration session each.

#### Human Relations Training Laboratory Group

Our search identified 1 paper testing the efficacy of combining a large dose of LSD with a Human Relations Training Laboratory (HRTL) group intervention in treating individuals suffering from AUD (Bowen et al., 1970). HRTL (Blake and Mouton, 1964) is a group training intervention built from the assumption that AUD patients struggle in dealing with everyday problems because of a lack of relational skills. The program therefore focuses on lectures on how to “increase effectiveness in groups” (Bowen et al., 1970), structured exercises and a rating system designed to assess progress. Patients were not attending any form of psychotherapy. The study (Bowen et al., 1970) described two experiments. In the first one a group of inpatients was assigned to a single LSD (500 μg, *n* = 41) session plus HRTL while a second one went through the HRTL program alone (*n* = 40). In the second experiment an additional group was included which went through a single LSD session (25 μg, *n* = ~22) plus HRTL. In this second experiment the 500 μg group comprised 22 patients and the HRTL only group 15. The LSD session took place during the first 3 weeks of the program in a room furnished informally with “potentially symbolic articles such as a flower, pictures and a two-sided mirror” where music chosen by the participant could be played. The session was preceded by “several group lectures to prepare patients” (Bowen et al., 1970) and during this preparation facilitators aimed at establishing positive expectations toward both the psychedelic experience and the clinical outcomes (Bowen et al., 1970). Patients were also encouraged toward relaxing and “going along” with the experience and were discouraged toward trying to control it (Bowen et al., 1970). LSD sessions were held by nursing assistants who were instructed to give close attention to patients, provide “positive suggestions” regarding their “ability to gain from the experience” (Bowen et al., 1970). Results showed no significant differences between any of the groups (Bowen et al., 1970).

#### MDMA-Assisted Therapy and Mindfulness for Adult Autistic Individuals

Our search identified 1 RCT investigating the potential of MDMA-ATM in the treatment of social anxiety in adult autistic individuals (Danforth et al., 2018). The treatment program followed a protocol published a couple of years earlier (Danforth et al., 2016).

The rationale for the treatment was built on the basis of (a) research mostly from the ‘60s and ‘70s indicating potential for clinical improvement (b) a better understanding of the importance of set and setting in ensuring the safety of patients (c) the copious anecdotal reports of MDMA-induced improvements in empathy, ease of communication, comfort in social settings, feelings of ease in one's own body reported in online fora (for a review see Danforth et al., 2016) and (d) evidence showing that practicing mindfulness skills has beneficial effects in social, emotional and cognitive areas of functioning of autistic individuals (Bögels et al., 2008; Spek et al., 2013). Concerning this latter point, the authors adapted the mindfulness skills training module from Dialectical Behavior Therapy (DBT), an already established model of psychotherapy (Linehan, 2018).

The model is structured in three phases as seen in other approaches. More specifically, before the first MDMA session, participants take part in three 60–90-min preparatory sessions (Danforth et al., 2018) aimed at building rapport, clarifying treatment structure, expected effect of the substance, at discussing relevant issues expressed by participants and practicing mindfulness. Furthermore, the training aims at providing patients with an enriched vocabulary useful to describe the psychedelic experience. Once the preparation phase is complete and before taking the MDMA, patients are involved in a guided progressive muscle relaxation exercise (McCallie et al., 2006) in a room furnished to facilitate feelings of comfort. Patients are then accompanied through the experience by two co-therapists (one male and one female) for the whole duration of the session. During its course, a variety of tasks are proposed including artistic activities, listening to music, writing, introspection and therapist interaction. Therapists should create and maintain a supportive and safe setting and discuss social challenges that patients may have. The MDMA session is followed by 4 non-drug sessions (after 1 day, 2 weeks, 1 month, and 6 months, respectively) during which details concerning the experience are gathered and discussed to facilitate the integration of the experience in everyday life (Danforth et al., 2018). A subsequent MDMA session with corresponding integration sessions then follows. Subsequently, patients are be involved in daily telephone check-ins. In a randomized, double-blind, placebo-controlled pilot study, 12 patients were assigned either to a MDMA-ATM (*n* = 8) or to a placebo plus mindfulness skills training group (*n* = 4). Both conditions comprised 3 preparatory sessions, 2 drug sessions (75 mg MDMA for the first session and 100 mg MDMA for the second or 2 inactive placebo) and 3 integration sessions after each drug session. The MDMA-ATM led to significantly greater improvements in social anxiety scores at the 6-month follow up and response rates (MDMA = 75%; control = 50%) (Danforth et al., 2018).

In summary, this model proposes 3 preparation/mindfulness training sessions, 2 MDMA sessions held by 2 therapists (1 female and 1 male) each preceded by a relaxation exercise and followed by 4 integration sessions. The psychotherapeutic method is adapted from the DBT mindfulness module, an already established model (Linehan, 2018).

#### Motivational Enhancement Therapy

Our search identified 5 papers on 2 separate studies investigating the efficacy of psychedelic-assisted MET in the treatment of AUD: 1 pilot RCT (Dakwar et al., 2020; Rothberg et al., 2021) and 1 open-label study (Bogenschutz et al., 2015, 2018; Nielson et al., 2018).

MET is a brief treatment model devised to achieve change in problem drinkers and individuals suffering from other addictive disorders (Miller, 1994). MET is based on the principles of motivational interviewing (Miller and Rollnick, 1991) and on the transtheoretical model of change (Prochaska, 1992) which conceptualizes the process of changing maladaptive behavioral patterns as a cycle comprising 6 stages, i.e., precontemplation, contemplation, determination, action, maintenance, and relapse, each requiring specific therapeutic actions (Prochaska and DiClemente, 1985, 1994). It begins with an initial structured assessment, resolves in 4 sessions held in a 90-day window and considers the mobilization of the clients' own resources as the primary mechanism for effective therapy. This model rests on earlier literature indicating that effective treatments for problem drinkers share some common principles (Orford, 1986): they provide feedback of personal risk or impairment, emphasize personal responsibility, give clear advice, provide alternative options for change, value an empathic attitude on the part of the therapist (as opposed to a more confrontational one) and promote clients' self-efficacy (Prochaska and DiClemente, 1985, 1994).

During precontemplation individuals are not considering change and are usually uninterested in initiating treatment. In contemplation, they begin to consider the problematic aspects of their behaviors and the costs/benefits of change. If correctly facilitated by the therapist, patients will reach the determination stage in which a firm stand to take action is established. Once patients begin to modify the problem behavior, they are considered to have reached the action stage. Should the new behavior pattern persist, after 3–6 months patients are considered to have reached the maintenance stage. Relapse is considered another stage of this cycle and, should patients experience such slips, they will be accompanied through the cycle again. During the first treatment session (Miller, 1994), patients receive feedback from the initial assessment phase which addresses addictive behaviors, symptoms, decisional considerations, and future plans. The second session takes place a week after the first one and is dedicated to build or strengthen patient motivation. The subsequent two sessions, held 4 and 10 weeks after the second one, focus on monitoring and encouraging change and its maintenance. Therapists are required to adopt 5 motivational principles in conducting sessions: express empathy, develop discrepancy, avoid argumentation, roll with resistance, support self-efficacy. More specifically, therapists should aim at building an empathic and collaborative relationship. In this frame, patients are considered the only ones who can decide to change and therapists as supportive consultants who listen rather than tell and build up rather than tear down. Since motivation is conceptualized as the result of increasing discrepancy between the current and desired state, therapists should help to develop such discrepancy in order to increase the chances of opening a discussion on change options. Argumentation is firmly discouraged in order to avoid the strengthening of defensive coping strategies. Additionally, the focus is on the consequences of addictive behaviors (as opposed to diagnostic aspects) and on leading clients themselves to be the ones voicing the arguments for change. Since ambivalence is viewed as a normal part of the treatment of addictive behaviors, therapists are encouraged to “roll with” resistance. Finally, the belief that clients can change their addictive behavior is crucial for therapeutic success, therefore therapists should directly adopt strategies to build and consolidate self-efficacy (Bandura, 1982).

MET was used in conjunction with psilocybin to treat AUD in a proof-of-concept study that recruited 10 patients (Bogenschutz et al., 2015). The intervention model spanned over the course of 12 weeks and employed a total of 7 MET sessions, 3 preparation sessions, 2 psilocybin sessions, and 2 debriefing sessions. More specifically, screened patients were involved in 2 preparation and 2 MET sessions followed by the first psilocybin session (0.3 mg/kg, p.o.). Subsequently, patients took part in a debriefing session, 2 more MET sessions and in another preparation session before proceeding to the second dosing session. In this case the proposed dose was 0.4 mg/kg unless the participant was unwilling to increase it, experienced adverse effects during the first one or already achieved a complete mystical experience. In these cases the employed dose would have been equal to that of the first session. The treatment course ended after another debriefing session and 3 more MET sessions. Psilocybin sessions took place in a living room-like space where patients were asked to lie down on a couch while listening to music through headphones and wearing eyeshades. Results showed that drinking and heavy drinking days decreased across weeks 5–12 compared to both baseline and 4-week since treatment initiation. Improvements were maintained at the 36-week follow-up. No data on response/remission rates was provided. Qualitative analysis of patient accounts of psilocybin sessions (Bogenschutz et al., 2018) revealed themes related to mystical experiences, feelings of forgiveness, self-compassion, acceptance and love as well as experiences of catharsis, increased mindfulness and “spaciousness.” According to the authors, the emerged material was personally meaningful to the individuals and tended to frame drinking behaviors in a wider psychodynamic perspective.

In another recent randomized, active-placebo controlled trial that compared the efficacy of a single ketamine hydrochloride (2-min 0.11-mg/kg bolus in saline followed by a 50-min slow-drip intravenous infusion of 0.6 mg/kg) or midazolam (a 2-min saline bolus followed by a 50-min slow-drip intravenous infusion of midazolam, 0.025 mg/kg) administration session combined with psychotherapy to treat AUD (Dakwar et al., 2020), MET was adapted as follows. During the course of 5 weeks, patients were involved in a course of weekly sessions. The initial session aimed at defining goals and elaborating a motivational statement related to quitting drinking. During the following week, they took part in an infusion session and in an additional MET session. The other sessions were scheduled for the following 3 weeks. Results showed better outcomes in the ketamine group in terms of drinking days, heavy drinking days, proportion of abstinent participants, at the 21 days post-infusion time point. Telephone interviews with a subset of the complete sample (*n* = 19) suggested greater rates of abstinence in the ketamine group (75%) compared to the control group (27%) at the 6 months follow-up. Additional analyses showed that the occurrence of mystical-type experiences seemed to play a role in mediating treatment efficacy (Rothberg et al., 2021).

In summary, the first adaptation (psilocybin) saw preparation and integration (debriefing) sessions interspersed in the course of a MET program with 1 preparation session and 1 debriefing session before and after each drug session. One extra preparation session was carried out at the beginning of treatment. The second adaptation (ketamine) used no formal preparation but included an MET motivational and goal-setting session before the infusion. Subsequently, 4 more MET sessions were administered.

## Discussion

The aim of the present work was to review the current therapeutic approaches adopted in clinical research on PAT to highlight common practices as well as diverging aspects and identify issues in need of development. While most of the evidence for the efficacy of PAT can be considered preliminary and the need for more placebo-controlled trials is clear, clinical outcomes in the investigated conditions (most of which are considered chronic and refractory to treatments) seem promising (i.e., reported response rates are between 57 and 88%; reported remission rates are between 18 and 85%), especially considering that improvements are clinically relevant, sustained, observed in a short window of time, with fewer drug administrations and talk-therapy sessions compared to more established therapeutic options. For instance, literature on the efficacy of antidepressants in individuals suffering from end-of-life depression shows no clear differences when compared with placebo (e.g., Ostuzzi et al., 2018). Regarding PTSD, the Committee on the Assessment of Ongoing Efforts in the Treatment of Posttraumatic Stress Disorder estimated that up to 50% of diagnosed patients can be classified as non-responders to first-line therapies (Institute of Medicine, 2014). Similar considerations on treatment resistance and risk of relapse can be made for TRD, heroin addiction and tobacco addiction (Krupitsky et al., 2002; Cahill et al., 2014; Carhart-Harris et al., 2018a). As apparent from the outcomes of the selection process (Figure 1), several studies were excluded because they did not provide descriptions of structured psychotherapeutic interventions. Most of these excluded studies did not report use of psychotherapy (e.g., several ayahuasca studies such as Palhano-Fontes et al., 2019 or the Grob et al., 2011 psilocybin study), provided only generic labels (e.g., *regular psychodynamic psychotherapy;* Müller et al., 2020) to refer to it (see for instance Berlowitz et al., 2019; Giovannetti et al., 2020) or referenced the Johnson et al. (2008) paper which actually provides guidelines for safety in the context of psychedelic clinical research (see for instance Griffiths et al., 2016; Davis et al., 2021). Regarding the final subset of included studies, despite the fact that proposed theoretical frameworks and treatment structures vary considerably, our review identified factors that are almost constant and still unresolved issues.

### Preparation

A preparation phase (varying in duration from 2 to 10 h) is almost always included in both adapted and *ad-hoc* models and its sessions are used for a variety of purposes. In summary, all but 9 reviewed studies (*n* = 43) explicitly mentioned a preparation phase. Almost all approaches use it to establish therapeutic alliance (Kolp et al., 2006; Johnson et al., 2014; Bogenschutz et al., 2015; Carhart-Harris et al., 2016; Danforth et al., 2016; Ross et al., 2016; Anderson et al., 2020; Dakwar et al., 2020; Jerome et al., 2020; Monson et al., 2020). In the case of TIMBER (Pradhan et al., 2017) and of a case report on ketamine plus CBT (Ocker et al., 2020), no details on preparation sessions were reported. In psilocybin plus CBT for smoking cessation (Johnson et al., 2014) and in the CBCT plus MDMA (Monson et al., 2020), no explicit mention of interventions to improve therapeutic alliance was made.

Preparation is almost always described as a setting to discuss issues relevant to the aims of the individual and the supposed therapeutic mechanism of the model. For instance, MAP recommends exploring the themes of diagnosis, prognosis and death while promoting connection with family members as a way to prevent isolation and cultivate meaningfulness (Grof and Halifax, 1978); KPT/KET, when applied to end-of-life anxiety, explores beliefs about death and the afterlife (Kolp et al., 2007); MDMA-AP reviews trauma history (Mithoefer, 2016); PSI and KPT/KET for TRD encourage patients to talk about their hypotheses concerning the origin of their depression, anxiety or addiction (Krupitsky and Grinenko, 1997; Carhart-Harris et al., 2016; Stroud et al., 2018); finally, all adapted approaches include a preliminary phase in which treatment rationale is explained and focus is placed on the aspects that need to be worked upon to achieve remission, wellbeing or improvement in social skills. Furthermore, approaches such as KPT/KET, MET and QFL also explore personal motives that led each patient to pursue treatment and may even require them to produce a motivational statement to be used during the psychedelic session(s) (Kolp et al., 2007; Johnson et al., 2014; Bogenschutz et al., 2015; Dakwar et al., 2020).

This phase has a lot in common with the concept of setting clear intentions that is central to the set and setting theory (Hartogsohn, 2017; Carhart-Harris et al., 2018b) and, while most approaches tend to assume a non-directive stance toward the themes to be discussed and the intentions and expectations to be set, there seems to be an exception in which therapist input plays a more central role. More on the directiveness end of the spectrum, in fact, we find KPT/KET whose preparatory sessions explicitly aim at structuring a therapeutic myth with considerable therapist input (Krupitsky and Grinenko, 1997). More specifically, positive expectations toward outcomes are established and patients are told that they will experience insights, that the causes of their addiction are unconscious in nature and related to their personality and that such causes will manifest themselves during the psychedelic session in symbolic form. Similar interventions, albeit described as less directive, can be found in the MDMA-AP treatment manual (Mithoefer, 2016). The authors, in fact, explicitly ask patients to trust their inner healing intelligence during the therapeutic process therefore assuming its “existence,” function and relevance. In both cases, whether such interventions are carried out with the aim of informing about an underlying process or to provide therapeutic suggestions to increase expectancy effects and, ultimately, therapeutic outcome is not explicitly addressed. This issue is especially relevant if we consider that psychedelics seem to increase suggestibility and, therefore, make patients more sensitive to the environment's influence, therapist included. Furthermore, we point out that most papers describing *ad-hoc* and adapted therapeutic approaches with the exception of KPT/KET and MDMA-AP, only provide brief descriptions of the information and suggestions provided during the preparation phase and therefore do not allow for an assessment of its potential suggestive effects. For instance, therapists should decide what aspect(s) should be discussed and therefore made salient in the preparation phases—be it the present symptoms, cognitions, behaviors, aspects related to conditioning processes, supposed causes, intrapsychic dynamics, existential issues or other clinically relevant themes. These considerations should stimulate clinicians and researchers alike to investigate the effects that such therapeutic strategies may have on outcomes and the most effective ways to frame PAT since the early preparation phases, to maximize effectiveness. In the case of CBCT (Monson et al., 2020) and MDMA-ATM (Danforth et al., 2016), preparation sessions are also used to train participants in acquiring skills that may be useful to go through the psychedelic experience and therapy in general.

### Drug Sessions

Drug sessions last between 45 min (i.e., ketamine sessions) and 8 h (i.e., psilocybin sessions), are always supervised by 1 or 2 clinicians, are often held in rooms that are decorated to resemble a living room-like environment rather than a medical office, often make use of music and eyeshades and, in some cases, contain objects of personal significance to the participant. Before the onset of the effects, intentions are often reiterated and participants are often encouraged to shift their attention inward. Based on the descriptions provided in the articles, when it comes to therapeutic stances during dosing sessions we noticed a continuum between two polarities. The first is the non-directive one (Grof and Halifax, 1978; Johnson et al., 2014; Bogenschutz et al., 2015; Carhart-Harris et al., 2016), in which therapists only aim at keeping the participants' attention inward and provide verbal and non-verbal support during challenging moments. These interventions include touching, rocking and holding hands as well as suggestions to use previously learned self-regulation techniques such as breathing and imagery exercises. It is the case of MAP, PSI, MDMA-ATM, psychedelic-assisted CBT, MDMA-assisted CBCT, and psilocybin-assisted MET (Grof and Halifax, 1978; Johnson et al., 2014; Bogenschutz et al., 2015; Carhart-Harris et al., 2016; Danforth et al., 2016; Monson et al., 2020). Moving toward more directive approaches, therapists in MDMA-AP are encouraged to identify avoidance strategies, direct patients' attention toward the issues that are considered relevant for the treatment and support them through the process (Mithoefer, 2016). Therapists are also required to remain receptive to hidden meanings that the contents of the session may suggest, thus promoting an actively interpretive stance. As was already apparent in the preparation phase, KPT/KET seems to adopt an even more directive stance. According to the original description of the method, during ketamine infusions, patients should be exposed to psychotherapeutic influences aiming at promoting sobriety and adaptive personality change (Krupitsky and Grinenko, 1997). Furthermore, the fact that this model incorporates spiritual, religious and/or transcendental elements to the therapy session may expose patients to the risk of incorporating the therapists' belief systems (Johnson, 2021). This issue is even more sensitive if we consider the increase in suggestibility that psychedelics seem to produce (Carhart-Harris et al., 2015). The same could be said for non-empirically verified notions concerning the action of psychedelics and the nature of constructs such as the mind, personality, the self and the specific conditions that patients may be suffering from Johnson (2021). Among those examined in this review, TIMBER is the most directive and structured one.

### Integration

Integration is a part of all approaches except for TIMBER (Pradhan et al., 2017) and is usually described as a phase in which the insights gained during the psychedelic experience are processed and generalized to everyday life. MAP, MDMA-AP, KPT/KET, and the psilocybin-assisted CBT model for smoking cessation consider the final phase of the drug session—in which the substance's effects are subsiding—as the beginning of the integration phase (Grof and Halifax, 1978; Krupitsky and Grinenko, 1997; Johnson et al., 2014; Mithoefer, 2016). Patients are encouraged to discuss their experience or to write down a report in an attempt to consolidate memories and promote integration to everyday life.

Most studies provide little information about the therapeutic stance held during the integration phase and here too we find coherence with some of the aspects we discussed above. For instance, MDMA-AP suggests the treatment as an ongoing process that unravels beyond the drug sessions themselves, into the integration sessions and in the patients' everyday life (Mithoefer, 2016). As is the case with PSI, SEGT and KPT/KEP, MDMA-AP also allows for occasional therapist interpretations of the psychedelic experience that are aimed at consolidating change and generating meaning (Krupitsky and Grinenko, 1997; Carhart-Harris et al., 2016; Mithoefer, 2016; Anderson et al., 2020). The inclusion of existentially oriented psychotherapies also seems to recur in this phase, supposedly because of its focus on meaningfulness (Grof and Halifax, 1978; Krupitsky and Grinenko, 1997).

### Directiveness, Information, Expectation and Suggestion

Early PAT models emphasized the importance of non-directiveness and of letting the therapeutic frame and the contents of sessions define themselves during the course of preparation, drug sessions, and integration (Grof and Halifax, 1978). This general direction was adopted by several more modern approaches and is apparent from both direct descriptions of the methods and the suggestion to integrate elements from therapeutic models derived from very different theoretical premises (e.g., supportive psychotherapy, cognitive-behavioral therapy, existentially oriented psychotherapies, psychodynamic psychotherapy, mindfulness-based treatments).

While this framing allows for considerable flexibility on the therapists' part, it also reveals that a clear picture of what may make PAT work is still missing. Since the first moments of preparation, patients are presented with information and suggestions concerning therapy that will establish specific expectations. This aspect should be thoroughly investigated, especially if we consider that expectations seem to play a relevant role in defining the effectiveness of clinical interventions (Muthukumaraswamy et al., 2021). In a more practical sense, therapists should know if, how and where they should orient the contents of the sessions and how to treat such materials once they surface. The studies mentioned in this review suggest that the focus could be placed on the supposed causes of suffering, present life, intrapsychic dynamics, existential issues, family issues and/or traumatic events.

Moving to the more interventionist contributions, some of them provide a structured model of the condition (e.g., lack self-regulation skills, maladaptive personality profiles), actively intervene to build a tailored therapeutic myth or suggest the presence of inner processes promoting healing that can act once patients surrender to the psychedelic experience. Considering the potential increase in suggestibility caused by psychedelic substances (Carhart-Harris et al., 2015), a better understanding of what works for whom is highly desirable and future research should focus on determining the general interventions that make PAT work and the specific adaptations that may be required for different patient populations. More concisely, therapists should know what aspects to make salient during the continuous process that is the establishment of appropriate therapeutic sets and settings.

Another way to look at the issue may be framing it in terms of potentially enhanced learning (Banks et al., 2021) and cognitive flexibility (Kuypers et al., 2016) that could facilitate acquisition and extinction. In this framework, psychedelics may be used to optimize already established CBT strategies in order to improve response rates and stability of results. Among the common themes emerged from our review we should also mention meaning-making as an aim of PAT. Aspects related to meaning making seem to be associated with psychological wellbeing (Preller et al., 2017) and psychedelic sessions have been found to increase perceived meaningfulness (Kaelen et al., 2018). Future research should focus on investigating how to integrate the search for meaning in PAT, in terms of the economy of both the single sessions and the therapeutic journey as a whole. Furthermore, while most approaches seem to value rapport, research on the potential moderating effects that varying degrees of therapeutic alliance may have on clinical outcomes of PAT is still in need of development (Carhart-Harris et al., 2018b). Finally, except for one study regarding KET/KPT (Krupitsky et al., 2007), we found no studies directly addressing the effects of treatment duration, number of drug and drug-free sessions on both clinical improvement and its stability in time. This is another relevant area to focus upon in future research.

## Conclusion

The present paper critically reviews the models of PAT framing to provide a comprehensive picture of current practices in clinical psychedelic research. While some structural aspects of PAT seem to recur in clinical studies, the therapeutic stance and theoretical frameworks seem far from being exhaustively defined. Considering that psychedelics seem to enhance sensitivity to the internal and external environment (i.e., suggestibility), future research should provide more details on how such environments are constructed in terms of suggestions, description of the mechanisms underlying conditions and treatments, setting of expectations, therapeutic models employed and quality of the therapeutic relationship. This review fills a gap in the current literature and provides a systematic way to think about psychotherapeutic framing of PAT. The concepts discussed above are relevant to future construction of studies, designing of training programs for aspiring psychedelic psychotherapists and are presented with the intention to contribute to the development and implementation of PAT in several fields of psychiatric, psychological and medical relevance.

## Data Availability Statement

The original contributions presented in the study are included in the article/supplementary material, further inquiries can be directed to the corresponding author.

## Author Contributions

MC and CM carried out the paper selection process. MC drafted the paper. All authors took part in the revision process and contributed to the article, design, interpretation of data, and approved the submitted version.

## Conflict of Interest

The authors declare that the research was conducted in the absence of any commercial or financial relationships that could be construed as a potential conflict of interest.

## Publisher's Note

All claims expressed in this article are solely those of the authors and do not necessarily represent those of their affiliated organizations, or those of the publisher, the editors and the reviewers. Any product that may be evaluated in this article, or claim that may be made by its manufacturer, is not guaranteed or endorsed by the publisher.
